# Differential Association of Glycation Products with Bone Mineral Density and Fat Mass in Healthy and Diabetes Type 2 Subjects from Mexican Southeastern: A Cross Sectional Study

**DOI:** 10.3390/medicina59081451

**Published:** 2023-08-11

**Authors:** Fernando Ferreyro-Bravo, Ángel Ceballos-Cruz, Mary Jose Urruchua-Rodríguez, Gabriela Martínez-Reyes, Carolina Cortés-Pastrana, Elda Leonor Pacheco-Pantoja

**Affiliations:** 1 Health Sciences PhD Program, Universidad Católica de Murcia UCAM, 30107 Guadalupe de Maciascoque, Murcia, Spain; faferreyro@alu.ucam.edu; 2 Health Sciences Division, School of Medicine, Anahuac Mayab University, Mérida 97308, Yuc., Mexico; iangelceballos@outlook.com (Á.C.-C.); maryjose.urruchua4@gmail.com (M.J.U.-R.); gabriela-martinez19@hotmail.com (G.M.-R.); caro_2297@hotmail.com (C.C.-P.)

**Keywords:** bone mineral density, glycation products, diabetes

## Abstract

*Background*: Glycation products have been linked to decreased bone mineral density (BMD) in a number of clinical settings. This study examined the correlation between early glycation products (HbA1c and glycated albumin (ALB-g)) and advanced glycation end products (pentosidine (PTD)) with BMD in two groups of participants: those with type 2 diabetes mellitus (DM2) and those without diabetes or any other comorbidities (noDM). All of the participants had resided in southeastern Mexico for a minimum of 10 years. *Material and Methods*: This study included 204 participants: 112 (55%) with DM2 and 92 (45%) healthy subjects. We utilized dual X-ray absorptiometry (DXA) to measure both the total and segment-specific BMD and adipose mass. In addition, the fasting blood glucose, HbA1c, PTD, and ALB-g parameters were measured. Correlation and logistic regression analyses were conducted. *Results*: There was an inverse correlation between PTD and BMD in all anatomical regions among postmenopausal women (PMW) in the DM2 group, whereas in non-PMW, only the waist-to-height ratio was statistically significant. A negative correlation was observed between HbA1c levels and BMD in the arms and legs of DM2 individuals. However, in the noDM group, a negative correlation was found between HbA1c levels and BMD in the pelvis, while a positive association was observed between HbA1c and indicators of adipose tissue. ALB-g, demonstrated a negative correlation with fat mass. After performing binary logistic regressions, the following odds ratios (OR) for osteopenia/osteoporosis risk were determined: PTD OR 1.1 (*p* = 0.047) for DM2 PMW, HbA1c OR 1.4 (*p* = 0.048), and fat mass content OR 1.011 (*p* = 0.023) for the entire sample. *Conclusions*: Glycation products are associated with BMD differentially depending on the analyzed anatomical segment, but PTD, HbA1c, and fat mass are significant predictors of low bone mass. In prospective studies, this association could be determined using other techniques involving three-dimensional analysis of bone architecture to evaluate bone architecture.

## 1. Introduction

The correlation between bone fragility and aging is a commonly acknowledged phenomenon that is influenced by chronic degenerative ailments such as diabetes, resulting in a reduction in bone mineral density (BMD) and bone mineral content (BMC) [1]. According to some reports, the presence of early glycation products (EGP) and advanced glycation end products (AGE) in the collagen of the bone matrix are potential factors that alter bone resistance [2]. The formation of EGPs and AGEs occurs via a sequence of non-enzymatic reactions between proteins and glucose or its derivatives, leading to the production of a remarkably stable end product. The majority of proteins are susceptible to the formation of AGEs; however, their accumulation is observed in tissues with a low rate of turnover. In this regard, collagen, for instance, manifests some modifications in its structure and functionality. According to the existing literature, it has been suggested that hyperglycemia, oxidative stress, and other pathological metabolic processes may be among the factors that contribute to glycation mechanisms [3].

Instances of interactions between these substances in humans include glycated hemoglobin (HbA1c) and glycated albumin (ALB-g) for EGP, and pentosidine (PTD) for AGE [4], which have been identified as potential outcome complication predictors in diabetes [5].

Research on the correlation between AGE, EGP, BMD, and BMC has yielded varying results, ranging from a statistically significant negative correlation [6] to a non-significant inverse correlation depending on the specific body segment being studied [7]. Consensus exists regarding the potential negative impact on bone metabolism, which is influenced by the specific comorbidity, gender, and age of the participants under investigation [8,9]. In this context, previous studies have reported a correlation between elevated levels of PTD and the likelihood of experiencing vertebral fractures in women aged 60 years and above, regardless of diabetic status. However, this association has not been observed in men [10,11]. On the other hand, López-Prieto et al. [12] observed a positive association between elevated serum levels of HbA1c and diminished bone mineral density (BMD) specifically in the lumbar region of the spine. This correlation maintained its statistical significance even when accounting for variables such as age, gender, and body mass index (BMI)1. Regarding ALB-g, although there is a lack of direct evidence establishing a causal link between elevated levels of ALB-g and compromised bone health, it can be inferred that such an association exists based on its potential as a biomarker for the detection of diabetes mellitus [13].

The relationship between EGP or AGE and fat mass remains uncertain, with some studies indicating an inverse correlation between serum circulating levels and obesity due to the retention of these substances within the adipose tissue [14,15].

The objective of this study was to examine the correlation between EGP markers (HbA1c and ALB-g) and AGE marker (PTD) with BMD, and fat mass in two groups of participants: those diagnosed with type 2 diabetes (DM2) and those without diabetes or any other comorbidities (noDM). Furthermore, all of the participants were individuals who had been residing in southeastern Mexico for at least 10 years. No studies were found that specifically examined the correlation between these compounds and bone mineral density or adipose tissue distribution in the Mexican population.

## 2. Materials and Methods

### 2.1. Study Subjects

The present cross-sectional investigation enrolled participants who had previously been diagnosed with DM2 (*n* = 112), as well as individuals who did not have diabetes or any other clinically evident comorbidities (noDM2) (*n* = 92). Uncompensated involvement was mandatory, and explicit and attested assent was necessary. The sampling methodology employed in this study involved issuing an open call for participation with a specified deadline. The sample was then selected from a list of registered volunteers categorized into the two groups mentioned before. The final participants were chosen using a systematic probabilistic sampling approach. The determination of the minimum sample size was carried out through the calculation of the sample size, in accordance with the methodology for assessing the statistical significance of a correlation coefficient where the assumptions comprised of a statistical power of 80%, an alpha value of 0.05 for two-tailed testing (representing the probability of a type I error), and an anticipated correlation coefficient of 0.3 (which is considered an acceptable correlation coefficient for a medium effect size) [16]. The present study underwent a thorough Ethics Review and was granted approval by the Clinical Research Ethics Committee of the responsible institution, School of Medicine, Anáhuac Mayab University, with a registry number of MED/083/18.

### 2.2. Demographic, Medical and Biochemistry Data Collection

Clinical data were collected by conducting interviews and recording the participants’ characteristics, including age, weight, height, time of initial diabetes diagnosis, and age at menopause onset (if applicable). The exclusion criteria encompassed individuals who were undergoing treatment for osteoporosis; engaged in smoking or alcohol consumption exceeding three units per week; were taking medication other than oral hypoglycemic prescription for diabetic subjects (such as insulin); had comorbidities or other complications such as retinopathy, neuropathy, and nephropathy; and weighed over 120 kg. It is important to note that given the average BMI for a person weighing 120 kg and having a height of 170 cm is approximately 40 kg/m^2^; we confirmed that those with a BMI greater than 40, who fell into the extreme obesity category, were also excluded from the study. The study involved obtaining fasting (for at least 8 h and no more than 12) venous blood, which was then subjected to various analytical determinations as follows: HbA1c content (turbidimetric inhibition immunoassay method, Cobas Roche Diagnostics GmbH, Penzberg, Germany), serum glucose, mg/dL (Glu; hexokinase enzymatic method, Cobas Roche Diagnostics GmbH, Penzberg, Germany), PTD concentration, ng/mL (enzyme-linked immunoassay [ELISA] method, Elabscience, Houston, TX, USA), and ALB-g concentration, mg/mL (ELISA method, DRG International, Springfield, NJ, USA). Briefly, the HbA1c method involved the binding of denatured HbA1c to latex particle antibody. The synthetic antigen-containing agglutinator inhibits latex agglutination by reacting with the HbA1c antibody. Concentration of HbA1c is determined by the inhibition of latex agglutination at 625 nm. HbA1c is expressed as a fraction of the total hemoglobin [17]. As for glucose determination, the enzymatic reference method is performed with hexokinase catalyzes the phosphorylation of glucose to glucose-6-phosphate by ATP [18]. PTD determination uses competitive-ELISA, which uses a PTD-coated micro ELISA plate. The reaction was competed with a predefined amount of PTD on the solid phase supporter for biotinylated detection Ab sites specific to PTD. The measurement was performed spectrophotometrically at 450 nm ± 2 nm [19]. ALB-g was detected utilizing an immobilized monoclonal antibody that specifically recognized the glycated moieties on human albumin. After a predetermined period, an enzyme-conjugated polyclonal antibody against human albumin was added. A chromogenic substrate was then added, and the color intensity was measured at 450 nm. A calibration curve determined the level of glycated albumin in a patient [20].

The body composition assessment included weight, height, waist circumference, and hip circumference. Additionally, four anthropometric scores were computed and documented: body mass index (BMI), waist-to-hip ratio (W/H), waist-to-height ratio (W/Ht), and relative fat mass (RFM) [21]. The participants’ height and weight were obtained using standardized techniques and instruments [22] and were conducted in adherence to the Official Mexican Norm [23], performed by a single researcher (F.F.B.), with a certification in kinanthropometry level 3 by The International Society for the Advancement of Kinanthropometry (ISAK). In addition, those parameters were acquired concurrently with the clinical interview utilizing a portable body composition monitor and scale (OMRON Healthcare Co., Ltd., Tokyo, Japan) on the same day. The measurements were taken without shoes and light clothing and were approximated to the nearest 0.1 kg. The Harpenden stadiometer (Holtain 602VR^®^, Crymych, UK) was utilized to measure height with a precision of 0.5 cm. Participants were instructed to remove their shoes during the measurement process. BMI was computed by dividing the weight in kilograms by the square of the height in meters (weight (kg)/height (m^2^)). The W/H and the W/Ht ratios were calculated correspondingly by dividing the measurement of waist girth divided by hip girth or height. RFM was calculated as indicated by Woolcot et al. [21] utilizing the metric of waist circumference ascertained at the anatomical location of the iliac crest. We incorporated diverse anthropometric indicators, such as BMI), W/H, W/Ht, and RFM, for the purpose of investigating their individual efficacy and correlation with the biochemical parameters that were under investigation.

### 2.3. Determination of BMD, BMC, and Fat Mass Distribution

The study employed dual X-ray absorptiometry (DXA; Hologic QDR Explorer, Madison Heights, MI, USA) to conduct measurements of BMD, BMC, and fat mass percentage in total and by-segments, including the arms, legs, spine, pelvis, and ribs. The accuracy of the instrument was assessed through duplicate measurements of BMD at the spine in a cohort of 30 participants. The margin of error for precision was determined to be 0.007 g/cm^2^, while the minimum detectable change was found to be 0.019 g/cm^2^ with a confidence level of 95%. The bone status categories were classified in accordance with the guidelines of the World Health Organization. These categories include normal bone status, which is defined as having a T-score greater than −1.0 SD; osteopenia, which is defined as having a T-score between −1.0 and −2.5 SD; and osteoporosis, which is defined as having a T-score of −2.5 or lower.

Subsequently, the participants were classified into two distinct categories, namely individuals with reduced bone density (comprising individuals with osteopenia−osteoporosis) and individuals with typical bone density (comprising individuals with normal bone density) to conduct binary logistic regression analysis.

The DXA evaluation was employed to assess the percentage of fat mass, as it has been consistently demonstrated to possess a satisfactory level of accuracy in measuring adiposity in individuals with a body mass index (BMI) exceeding 18.5 kg/m^2^ [24].

### 2.4. Statistical Analysis

The Shapiro−Wilk (S-W) test was employed to analyze the probability distribution of the variables. The statistical analysis involved a two-sided Student’s *t*-test to examine quantitative variables that exhibited a normal distribution. The Mann−Whitney U test was utilized to conduct nonparametric comparisons of variables. The Spearman linear correlation coefficient was employed to calculate the bivariate correlation of quantitative variables [25]. Using the values of HbA1c, ALB-g, and PTD, and the densitometric analysis (bone and fat mass) values of each anatomical segment, correlation analyses were undertaken per group (diabetic subjects versus non-diabetic subjects for each gender and menopausal status). When applicable, descriptive results are expressed as percentages, means, medians, standard deviations, or interquartile ranges.

For the logistic regression analysis, the variable that was subject to change based on the classification was the level of bone mineral density, which was categorized as either “low” (osteopenia/osteoporosis collapsed) or “normal”. The analysis included the co-variables of PTD, ALB-g, HbA1c, and fat mass. The examination was conducted on individual subgroups based on their metabolic status (diabetes mellitus and non-diabetes mellitus), gender, and menopausal classification.

Statistical significance was set at *p* < 0.05. SPSS^®^ version 24.0 for IOS (SPSS Inc., Chicago, IL, USA) was utilized. Laboratory analytic procedures were performed twice by blinded different people, DXA measurements were conducted by a trained technician, and anthropometric parameters were recorded by a certified professional in kineanthropometry. The statistical analyses were conducted for a blinded researcher who did not know whether the patients were healthy or diabetic until the final data integration. There were no missing values detected in the dataset.

## 3. Results

### 3.1. Demographic and Clinical Characteristics

Table 1 contains the descriptive data of the study subjects. The total number of participants was 204, with 112 (55%) diagnosed with DM2 and 92 (45%) without evident comorbidities; there was no significant difference in age (range 58, min 25, max 83) between the two groups. The significant differences between the two groups in the glycemic control parameters (Glu, *p* < 0.001; HbA1c, *p* < 0.001), and anthropometric measures such as waist-hip ratio (*p* = 0.007) and BMI (*p* = 0.04), confirmed the diabetic status of the patients. However, the percentage of total body fat mass (measured by DXA) did not differ statistically between the two groups.

### 3.2. Correlation Analyses between BMD, BMC, Fat Mass, and Biochemical Determinations

The findings were systematically arranged to display the correlation coefficients and corresponding *p*-values for every subgroup, including DM2 and noDM, gender, and menopausal status. The study conducted analyses utilizing DXA measurements for each segment, specifically evaluating BMD, BMC, and fat mass, as well as calculated scores such as BMI, waist/hip, waist/height, RFM, and girth measurements, in conjunction with either PTD, HbA1c, or ALB-g.

The outcomes for the entire sample are presented in Table 2, indicating that the sole noteworthy findings for PTD were observed in the spine region for both BMD (*p* = 0.049) and BMC (*p* = 0.014), displaying an inverse association. A negative correlation was observed for fat in the trunk (*p* = 0.045) and ALB-g, and a positive correlation between HbA1c and certain anthropometry scores, including BMI (*p* = 0.009), RFM (*p* = 0.005), W/H (*p* = 0.014), W/Ht (*p* = 0.003), and waist girth (*p* = 0.013).

#### 3.2.1. Correlations for the Diabetic and Non-Diabetic Categories

The results for DM2 and noDM categories are displayed in Table 3. The DM2 group demonstrated significant negative correlations between PTD and BMD or BMC in almost all of the segments, except for ribs BMD. The only significant negative correlation between fat mass (DXA) and PTD was observed for the thorax (*p* = 0.006). Nevertheless, anthropometric measurements exhibited substantial negative correlations for most of the measurements, except for the hip circumference. Consequently, BMI (*p* = 0.023), W/Ht (*p* = 0.020), RFM (*p* = 0.017), W/H (*p* = 0.043), waist (*p* = 0.005), and neck (*p* = 0.004) circumferences were negatively associated with PTD.

ALB-g exhibited a negative correlation with the total BMC in the noDM group (*p* = 0.040). For the DM2 group, this EGP demonstrated an inverse relationship with trunk fat (g) (*p* = 0.007), as well as BMI (*p* = 0.030) and hip girth (*p* = 0.005).

Interestingly, HbA1c exhibited negative correlations with BMD and BMC limbs (arms and legs), but positive correlations with fat (%) in all measurements (trunk, legs, and arms).

We then analyzed the gender-specific correlations between DM and noDM, and the results are described in the following sections.

#### 3.2.2. Correlations for the Category of Men

Table S1 Supplementary, exclusively displays the statistically significant correlations pertaining to the DM male group, as the noDM men did not show any significant results. The analysis yielded a reduced number of statistically significant associations, wherein PTD exhibited positive correlations for RFM (*p* = 0.037) and BMI (*p* = 0.021). The results indicated an inverse correlation between HbA1c and BMC in arms (*p* = 0.037) and BMD pelvis (*p* = 0.021). Additionally, a moderate yet significant positive correlation was observed for fat (g) in legs (*p* = 0.045).

#### 3.2.3. Correlations for the Category of Women

Table S2 Supplementary presents the correlations related to females with and without DM2. The findings suggest that there an inverse association exists between PTD and BMD and BMC in all anatomical regions. In addition, it demonstrates noteworthy negative correlations with the quantities of adipose tissue present in the trunk and legs, along with BMI, waist-to-height ratio, and the three circumferences within the DM2 cohort. The study found that ALB-g exhibited negative correlations exclusively with total BMC (*p* = 0.015) and arms BMC (*p* = 0.039) in the DM2 group. Furthermore, the results indicated that there is an inverse relationship between HbA1c and both the BMD and BMC of the arms, with statistical significance at *p* = 0.003 and *p* = 0.012, respectively. Additionally, there is a positive correlation between HbA1c and the percentage of fat mass measured by DXA (%) across all body segments in the DM2 group.

#### 3.2.4. Correlations in Post-Menopausal and Non-Post-Menopausal Women in the DM2 Cohort

We then conducted a menopausal status classification analysis on the women in DM2. Table 4 displays the outcomes of the aforementioned correlations. The findings indicate that the inverse associations between BMC and BMD were observed in nearly all anatomical regions among postmenopausal women (PMW), whereas solely the waist-to-height ratio exhibited statistical significance among non-menopausal women (noPMW). Regarding ALB-g, correlations were mostly for fat mass by DXA (%) in the PMW category, with only two significant correlations for BMC limbs (*p* = 0.030) and spine (*p* = 0.007). Surprisingly, HbA1c was only negatively associated with arm BMD (*p* = 0.021) and positively associated with fat mass by DXA (g and %) in legs (*p* = 0.048 and *p* = 0.004, respectively).

### 3.3. Binary Logistic Regression

Within subgroups, we evaluated models that incorporated the PTD, ALB-g, HbA1c, and lipid content. Table 5 displays the only two models with a statistically significant result and a reasonable fit, as indicated by *p*-values less than 0.05 for the omnibus test and greater than 0.05 for the Hosmer−Lemeshow test. HbA1c (*p* = 0.048) and fat content (*p* = 0.023) were identified as significant predictors. In the case of HbA1c, odds ratio suggest that diabetics are 1.4 times more likely to have inadequate bone mineral density. The beta coefficient for fat content (g) only attained 1.011, notwithstanding its statistical significance.

Our analysis revealed that PTD remained statistically significant (*p* = 0.047) in females, with an odds ratio of 1.2, indicating that females in this group may be more susceptible to conditions characterized by reduced bone density, such as osteoporosis and osteopenia.

## 4. Discussion

This study provides valuable insights into the relationship between bone mineral density (BMD), adipose content, and biochemical markers in a sample of individuals from the southeastern Mexican region with and without type 2 diabetes mellitus. Age differences between the categories were not statistically significant, indicating that differences in age did not influence the observed results. Nevertheless, significant differences were observed in glycemic control parameters (glucose and HbA1c) and anthropometry indicators (waist-hip ratio and BMI), confirming the diabetic status of the patients. One of the significant and key results was the inverse correlation between PTD and BMD and BMC in the spinal region, as indicated by the overall correlations calculated for the entire study population. In this regard, a recent systematic review [26] discovered that pentosidine exhibited a direct association with both bone parameters and fracture risk in the available literature. In this study, it was determined that the existing evidence is consistent and points to an increased fracture risk in subjects with high levels of pentosidine. Furthermore, the correlation between PTD and BMD in diabetic patients was reported in only seven out of the forty-three final publications analyzed. Only three of those reported significant findings.

In our study, DM2 postmenopausal women were found to have significant negative correlations for all of the anatomical regions examined. In fact, this association has been reported more frequently in females, and Japanese postmenopausal women have demonstrated a significant association between fracture risk and PTD [7,10]. However, in the study reported by Nakano et al. [7], PTD was detected in the urine. However, they suggested that PTD may be a may be a risk for fractures independent of other traditional indicators, such as age, BMD itself, and previous fractures. In fact, our logistic regression model revealed that PTD was associated with a 1.1-fold increased risk of decreased bone mass in this cohort.

No statistically significant correlation was found between BMD and PTD in the male subjects. Moreover, the current body of literature offers limited empirical support regarding the interplay of these variables in males. One study, albeit with a small sample size, reported a negative association between PTD and both femoral strength and femoral BMD [27]. In a study focused on males diagnosed with type 2 diabetes mellitus (DM2), it was observed that these individuals exhibited elevated ratios of the PTD receptor in relation to PTD free, which were found to be associated with a reduced risk of fractures [28]. The aforementioned observations may potentially give rise to conjecture that PTD has an affinity for collagen fibers in certain individuals.

The role of adipose tissue in relation to PTD remains uncertain; however, it has been suggested that adipose tissue may possess the ability to retain PTD, resulting in reduced levels of PTD in the bloodstream [15,29]. When we analyzed our data, it was determined that there was no statistically significant correlation between indicators of fat content and PTD across the entire sample. However, when we analyzed the diabetic group, these variables exhibited significant inverse correlations; this phenomenon may be attributed to the detrimental accumulation of PTD, which could potentially modulate or hinder a signal originating from adipose tissue. One such signal is adiponectin, which is recognized as a protective factor in type 2 diabetes (DM2) [30] and has been found to impact markers related to bone formation [31].

In fact, the logistic regression model, which incorporated PTD as a variable, demonstrated that fat mass, as determined by DXA in grams, was a statistically significant predictor of low bone mass, albeit with a modest effect size (OR 1.011). Nonetheless, previous studies have indicated that individuals with a high BMI exhibit notably reduced serum pentosidine levels, which may be attributed to a potential decline in nitric oxide [32]. In our sample, DM2 women exhibited significant inverse correlations with anthropometric indexes. This finding suggests a differential and distinctive accumulation of adipose tissue in our diabetic subjects compared with healthy subjects, as previously reported [33,34], with implications for differential signaling of adipokines towards the skeleton [31]. For the DM2 group of men, there were positive correlations between PTD and both BMI and RFM, whereas in the noDM group, there were no significant results. In contrast with our findings, an aforementioned study [32] discovered a negative correlation in males between PTD and BMI when they divided the sample by gender; however, they studied only healthy subjects.

On the other hand, it was observed that individuals with inadequate glycemic control exhibited decreased serum levels of 25-hydroxyvitamin D and osteocalcin, both of which serve as biochemical markers for bone formation. This finding suggests that HbA1c may function as an independent risk factor for these specific metabolites [35]. In our study, we observed a negative correlation between HbA1c and BMD in both groups of participants. However, the patterns of this correlation differed between the groups. In the DM2 group, the negative correlation was observed for the arms and legs, whereas in the noDM group, it was observed for the pelvis. Indeed, the OR for HbA1c as a predictor was 1.4 for the entire sample in a model that included the three studied glycation products and fat mass. Additionally, we found that the levels of HbA1c in blood were positively associated with indicators of adipose tissue, as measured by DXA or anthropometry, in the entire sample, as well as in specific subgroups such as all DM2 participants, DM2 men, DM2 women, and DM2 postmenopausal women. These findings are consistent with previous published research [36].

Regarding ALB-g, the primary observations indicated a negative correlation with fat (DXA), both in grams (for the entire sample and DM2 group) and as a percentage in DM2 postmenopausal women. The inverse relationship between ALB-g and fat mass has been established in previous studies [37,38], and it has been recognized as a reliable measure for monitoring glycemic control in individuals with diabetes over a moderate period of time [39]. In the present study, we observed that postmenopausal women with diabetes exhibited elevated levels in circulation, as shown in Figure S1 Supplementary (only included for the discussion section).

In brief, even though the findings of our study suggest a correlation between EPG or AGE and BMD in individuals with diabetes, the extent of this association remains uncertain due to existing literature, indicating that osteoporotic fractures can occur in diabetic patients even in the absence of noticeable changes in bone density, a phenomenon referred to as the “diabetic paradox” [40].

It is acknowledged that the cross-sectional design and the sample size employed in our study lack the ability to establish causality definitively, thus recognizing the limitations in the generalizability of our findings. It is essential to interpret the results of this study with caution, recognizing that future research with a larger cohort is necessary to corroborate and further explore our findings.

Despite these limitations, the results underscore the significance of adopting more holistic methodologies for investigating bone homeostasis and mitigating progressive degenerative conditions such as osteoporosis.

## 5. Conclusions

Our results indicate that there is an association between EPG or AGE and BMD in diabetic subjects; however, the extent of this association is not entirely clear, as there is published evidence that osteoporotic fractures can be prevalent, even in the absence of obvious densitometric alterations.

The relationship between EPG and AGE with adipose mass or BMD in individuals with diabetes is clearly differentiated. We are inclined to suggest that compromised bone architecture arises from the accumulation of PTD within the bone matrix, in conjunction with adipose tissue involvement.

## Figures and Tables

**Table 1:** Characteristics of the participating subjects.

	DM2		noDM		Total	*p*
Participants, *n* (%)	112 (55%)		92 (45%)		204 (100%)	
	Men	Women	Men	Women		
Demographics						
Gender, *n* (%)	46 (22.5%)	66 (32.4%)	18 (8.9%)	74 (36.2%)	204 (100%)	n.s.*
Age, years, median (i.q.)	50 year (11)		55.5 year (13)		57 year (13.5)	n.s.*
Menopausal age, median (i.q.) *n* (%)	N.A.	48 year (10) *n* = 54 (26.5%)	N.A.	50 year (5) *n* = 56 (27.5%)	50 year (6.3) *n* = 55 (54%)	n.s.
DM2 time duration median (i.q.)	10 (16.3)		N.A.		10 (16.3)	
Body composition parameters						
BMI mean (s.d.)	30.9 (5.3)		27.7 (4.8)		29.4 (5.3)	0.005
W/H ratio mean (s.d.)	0.93 (0.08)		0.88 (0.07)		0.91 (0.8)	0.004
W/Ht ratio mean (s.d.)	0.62 (0.08)		0.57 (0.07)		0.60 (0.08)	0.004
Total Fat percentage mean (s.d.)	40.9 (6.8)		40.8 (8.2)		40.9 (7.4)	n.s.
BMD categories (WHO criteria) (% from each group)						
Normal, *n* (%)	40 (71.4%)		34 (73.9%)		74 (72.5)	n.s.
Osteopenia, *n* (%)	15 (26.8%)		11 (23.9%)		26 (25.5)	n.s.
Osteoporosis, *n* (%)	1 (1.8%)		1 (2.17%)		2 (1.9)	n.s.
Biochemical tests						
Glycemia mean, mg/dL (s.d.)	161.7 (61.5)		98.5 (21.9)		133.5 (57)	<0.001
HbA1c mean, % (s.d.)	7.7 (1.94)		5.4 (0.32)		6.6 (1.8)	<0.001
Serum PTD median, ng/mL (i.q.)	12.7 (24.1)		6.0 (30.3)		8.21 (26.4)	n.s.
Serum ALB-g median, mg/mL (i.q.)	2.5 (2.9)		2.8 (4.3)		2.7 (3.3)	n.s.

BMI: body mass index; HbA1c: glycated hemoglobin; BMD: bone mineral density; WHO: World Health Organization; s.d.: standard deviation, i.q.: interquartile range, WH: waist-to-hip, W/Ht: waist/height, n.s.: non-significant, N.A. not applicable, PTD: serum pentosidine, ALB-g: glycated albumin. Percentages are calculated from the total sample except when noted. * Proportion of diabetic men or women vs. non-diabetic men or women, respectively.

**Table 2:** Coefficient correlations for the whole sample (*n* = 204).

PTD				ALB-g				HbA1c		
		r	*p*			r	*p*		r	*p*
BMD Spine		−0.216	0.049	Fat Trunk (g) (DXA)		−0.218	0.045	BMI	0.281	0.009
BMC Spine		−0.269	0.014					RFM	0.300	0.005
								W/H	0.265	0.014
								W/Ht	0.322	0.003
								Waist girth	0.268	0.013

PTD = pentosidine; ALB-g = glycated albumin; HbA1c = glycated hemoglobin; BMD = bone mineral density; BMC = bone mineral content; RFM = relative fat mass; BMI = body mass index; W/H = waist-to-hip ratio; W/Ht = waist-to-height ratio; DXA= dual X-ray absorptiometry.

**Table 3:** Correlation coefficients between ALB-g, HbA1c and PTD in DM2 (*n* = 112) and noDM (*n* = 92) groups (females and males).

		DM2		noDM		DM2		noDM		DM2		noDM	
		PTD				ALB-g				HbA1c			
		r	*p*	r	*p*	r	*p*	r	*p*	r	*p*	r	*p*
BMD (g/cm^2^)	Total	−0.272	0.099	0.168	0.27	−0.017	0.910	−0.216	0.190	**−0.372**	**0.022**	−0.060	0.680
	Arms	**−0.381**	**0.018**	0.125	0.410	0.030	0.840	−0.191	0.250	**−0.479**	**0.002**	0.028	0.850
	Ribs	−0.270	0.102	0.072	0.640	0.095	0.520	−0.302	0.060	−0.248	0.130	0.276	0.060
	Spine	**−0.401**	**0.013**	−0.092	0.540	−0.091	0.540	−0.059	0.720	−0.200	0.230	−0.167	0.260
	Pelvis	**−0.402**	**0.012**	0.136	0.370	0.091	0.540	−0.147	0.370	0.055	0.740	**−0.345**	**0.018**
	Legs	**−0.436**	**0.006**	0.193	0.200	0.024	0.870	−0.234	0.160	**−0.376**	**0.020**	−0.104	0.480
BMC (g)	Total	**−0.432**	**0.007**	0.172	0.250	−0.080	0.610	**−0.340**	**0.040**	**−0.420**	**0.009**	−0.106	0.470
	Arms	**−0.441**	**0.006**	0.134	0.380	−0.150	0.320	−0.307	0.060	**−0.418**	**0.009**	−0.152	0.310
	Ribs	**−0.372**	**0.021**	0.167	0.270	0.060	0.710	−0.080	0.640	−0.225	0.170	0.229	0.120
	Spine	**−0.540**	**0.000**	−0.081	0.590	−0.110	0.470	−0.220	0.190	−0.244	0.140	−0.130	0.380
	Pelvis	**−0.379**	**0.019**	0.152	0.310	−0.020	0.900	−0.190	0.250	−0.291	0.080	**−0.303**	**0.038**
	Legs	**−0.499**	**0.001**	0.213	0.150	−0.020	0.880	−0.310	0.060	**−0.417**	**0.009**	−0.115	0.440
Fat mass (g)	Total	−0.294	0.070	0.168	0.270	**−0.316**	**0.030**	−0.027	0.870	0.165	0.320	−0.132	0.370
	Arms	−0.060	0.720	0.187	0.210	−0.186	0.210	−0.040	0.810	0.156	0.350	−0.108	0.460
	Trunk	**−0.440**	**0.006**	0.067	0.660	**−0.387**	**0.007**	−0.112	0.500	0.124	0.460	−0.134	0.370
	Legs	−0.202	0.220	0.243	0.100	−0.110	0.460	−0.110	0.510	0.302	0.060	−0.245	0.090
Fat mass (%)	Total	−0.069	0.680	−0.010	0.940	−0.024	0.870	−0.066	0.690	**0.404**	**0.012**	−0.091	0.540
	Arms	0.154	0.350	0.024	0.870	−0.050	0.730	−0.076	0.640	**0.342**	**0.036**	−0.056	0.710
	Trunk	−0.136	0.410	−0.046	0.760	−0.050	0.730	−0.006	0.970	**0.326**	**0.046**	−0.163	0.270
	Legs	0.103	0.540	−0.028	0.850	−0.022	0.880	−0.060	0.720	**0.482**	**0.002**	−0.134	0.360
Anthropometry Indexes	BMI	**−0.368**	**0.023**	0.248	0.100	**−0.324**	**0.030**	−0.029	0.860	0.122	0.460	0.004	0.970
	W/Ht	**−0.375**	**0.020**	0.189	0.210	−0.196	0.180	0.045	0.780	0.194	0.240	0.137	0.360
	RFM	**−0.384**	**0.017**	0.013	0.930	−0.172	0.240	−0.066	0.690	0.144	0.380	−0.064	0.670
	W/H	**−0.331**	**0.043**	0.180	0.230	−0.005	0.970	0.190	0.250	0.084	0.620	0.135	0.360
	Waist girth	**−0.446**	**0.005**	0.213	0.160	−0.225	0.130	−0.108	0.520	−0.007	0.960	−0.008	0.960
	Hip girth	−0.308	0.060	0.150	0.320	**−0.400**	**0.005**	−0.149	0.370	−0.034	0.840	−0.174	0.240
	Neck girth	**−0.459**	**0.004**	0.119	0.430	−0.257	0.080	−0.096	0.570	−0.118	0.480	−0.107	0.470

DM2 = diabetes mellitus 2; noDM = no diabetes mellitus; PTD = pentosidine; ALB-g = glycated albumin; HbA1c = glycated hemoglobin; BMD = bone mineral density; BMC = bone mineral content; RFM = relative fat mass; BMI = body mass index; W/H = waist-to-hip ratio; W/Ht = waist-to-height ratio.

**Table 4:** ALB-g, HbA1c, and PTD correlations for DM2 post-menopausal women (*n* = 54) and non-menopausal (*n* = 12) groups.

		noPMW		PMW		noPMW		PMW		noPMW		PMW	
		PTD				ALB-g				HbA1c			
		r	*p*	r	*p*	r	*p*	r	*p*	r	*p*	r	*p*
BMD (g/cm^2^)	Total	0.322	0.243	**−0.602**	**0.002**	0.041	0.884	−0.089	0.687	−0.469	0.078	−0.002	0.994
	Arms	−0.084	0.766	**−0.459**	**0.028**	0.211	0.449	−0.168	0.443	−0.154	0.584	**−0.477**	**0.021**
	Ribs	−0.180	0.521	**−0.442**	**0.035**	0.181	0.519	−0.275	0.204	−0.254	0.360	0.082	0.710
	Spine	−0.213	0.446	**−0.516**	**0.012**	0.458	0.086	−0.228	0.295	−0.061	0.830	0.156	0.476
	Pelvis	−0.178	0.526	**−0.494**	**0.017**	0.286	0.301	−0.199	0.362	0.077	0.785	0.268	0.217
	Legs	−0.160	0.568	**−0.642**	**0.001**	0.136	0.63	−0.142	0.519	−0.243	0.383	−0.063	0.776
BMC (g)	Total	−0.099	0.725	**−0.562**	**0.005**	0.371	0.173	−0.394	0.063	−0.061	0.830	−0.220	0.312
	Arms	−0.252	0.364	**−0.432**	**0.040**	0.154	0.585	**−0.453**	**0.030**	0.132	0.639	−0.383	0.072
	Ribs	−0.121	0.668	−0.409	0.053	0.504	0.056	−0.262	0.227	0.196	0.483	−0.171	0.437
	Spine	−0.245	0.379	**−0.590**	**0.003**	0.421	0.118	**−0.548**	**0.007**	0.014	0.960	0.002	0.993
	Pelvis	−0.016	0.954	**−0.464**	**0.026**	0.339	0.216	−0.228	0.295	−0.132	0.639	0.108	0.623
	Legs	−0.261	0.347	**−0.602**	**0.002**	0.357	0.191	−0.377	0.076	−0.004	0.990	−0.274	0.205
Fat mass (g)	Total	−0.258	0.354	−0.275	0.205	0.336	0.221	−0.279	0.198	0.332	0.226	0.323	0.133
	Arms	−0.159	0.572	−0.228	0.295	0.104	0.713	−0.222	0.308	0.239	0.390	0.169	0.442
	Trunk	−0.441	0.099	**−0.439**	**0.036**	0.321	0.243	−0.343	0.109	0.214	0.443	0.202	0.356
	Legs	−0.135	0.631	−0.393	0.063	0.271	0.328	−0.385	0.070	0.289	0.296	**0.407**	**0.048**
Fat mass (%)	Total	0.148	0.599	−0.208	0.342	0.193	0.491	**−0.517**	**0.012**	0.389	0.152	0.355	0.097
	Arms	0.209	0.455	0.101	0.647	0.054	0.850	**−0.443**	**0.034**	0.250	0.369	0.284	0.189
	Trunk	0.341	0.214	−0.208	0.342	0.211	0.451	**−0.431**	**0.040**	0.325	0.237	0.179	0.413
	Legs	0.276	0.320	−0.282	0.193	0.088	0.756	**−0.471**	**0.023**	0.154	0.584	**0.576**	**0.004**
Anthropometry Indexes	BMI	−0.496	0.060	−0.299	0.165	0.182	0.516	−0.125	0.571	0.089	0.752	0.272	0.209
	W/Ht	**−0.563**	**0.029**	−0.313	0.146	0.052	0.854	−0.014	0.948	0.132	0.638	0.289	0.181
	RFM	−0.465	0.081	−0.262	0.227	0.196	0.483	−0.178	0.417	0.461	0.084	0.290	0.180
	W/H	−0.454	0.089	−0.156	0.477	0.284	0.304	0.337	0.115	0.270	0.330	0.075	0.733
	Waist girth	−0.482	0.069	−0.367	0.085	0.177	0.528	−0.200	0.360	0.093	0.742	0.126	0.566
	Hip girth	−0.395	0.146	−0.283	0.191	0.111	0.694	−0.352	0.100	0.046	0.869	0.093	0.671
	Neck girth	−0.487	0.066	−0.309	0.152	0.043	0.879	0.009	0.966	−0.007	0.98	0.082	0.709

PMW = postmenopausal women; noPMW = non-menopausal women; DM2 = diabetes mellitus 2; noDM = no diabetes mellitus; PTD = pentosidine; ALB-g = glycated albumin; HbA1c = glycated hemoglobin; BMD = bone mineral density; BMC = bone mineral content; RFM = relative fat mass; BMI = body mass index; W/H = waist-to-hip ratio; W/Ht = waist-to-height ratio.

**Table 5:** Binary logistic regression models.

Model	Variables	Odds Ratio	95% CI	*p*
Entire sample Omnibus test of model coefficients: 0.031 Hosmer-Lemeshow test: 0.113	PTD	1.001	0.949–1.057	0.963
	ALB-g	0.847	0.592–1.211	0.362
	HbA1c	1.443	1.005–2.093	**0.048**
	Total fat mass (g)	1.011	1.001–1.021	**0.023**
Diabetic post-menopausal women Omnibus test of model coefficients: 0.018 Hosmer-Lemeshow test: 0.775	PTD	1.104	1.010–1.223	**0.047**
	ALB-g	0.526	0.246–1.125	0.098
	HbA1c	1.416	0.790–2.540	0.243
	Total fat mass (g)	1.000	0.999–1.000	0.082

## Data Availability

The datasets generated and/or analyzed during the current study are available from the corresponding author upon reasonable request.

## Supplementary Materials

The following supporting information can be downloaded at: https://www.mdpi.com/article/10.3390/medicina59081451/s1, Figure S1: Comparison of serum ALB-g levels in DM2 women, by PMW and noPMW categories; Table S1: Correlation coefficients for PTD and HbA1c in DM2 men; Table S2: ALB-g, HbA1c, and PTD correlations in DM and noDM women.
