# Supplementary material for: Differential Association of Glycation Products with Bone Mineral Density and Fat Mass in Healthy and Diabetes Type 2 Subjects from Mexican Southeastern: A Cross Sectional Study
Source: Medicina (Kaunas). 2023 Aug 11;59(8):1451. doi: 10.3390/medicina59081451 (PMC10456706; doi:10.3390/medicina59081451)
Supplement: Supplementary file 1 [file medicina-59-01451-s001.zip › Figure S1 suppl.pdf]

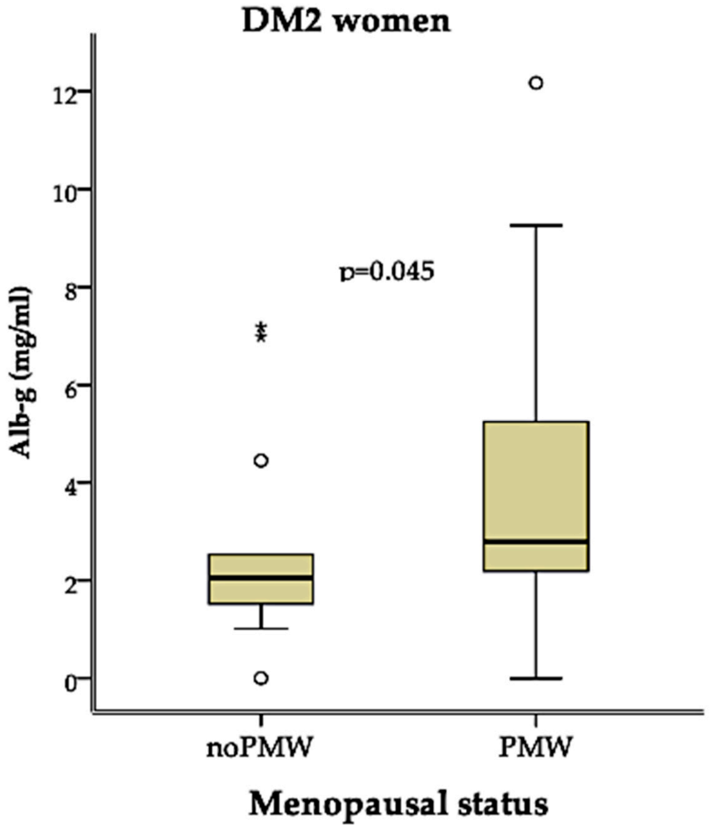

Figure S1. Comparison of serum ALB-g levels in DM2 women, by PMW and noPMW categories (ALB-g= glycated albumin, DM2=Diabetes mellitus 2, PMW= Postmenopausal women, noPMW=Non-postmenopausal women)
