# Supplementary material for: Differential Association of Glycation Products with Bone Mineral Density and Fat Mass in Healthy and Diabetes Type 2 Subjects from Mexican Southeastern: A Cross Sectional Study
Source: Medicina (Kaunas). 2023 Aug 11;59(8):1451. doi: 10.3390/medicina59081451 (PMC10456706; doi:10.3390/medicina59081451)
Supplement: Supplementary file 1 [file medicina-59-01451-s001.zip › Table S1 suppl.pdf]

Table S1. Correlation coefficients for PTD and HbA1c in DM2 men (n=46).

PTD			HbA1c		
	r	p		r	p
RFM	0.482	0.037	BMC Arms	-0.505	0.027
BMI	0.525	0.021	BMD Pelvis	-0.525	0.021
			Fat mass Legs (g)	0.464	0.045

PTD=Pentosidine, HbA1c= glycated hemoglobin, RFM= Relative fat mass, BMI= Body mass index, BMD= Bone mineral density, BMC=Bone mineral content.
