# Supplementary material for: Differential Association of Glycation Products with Bone Mineral Density and Fat Mass in Healthy and Diabetes Type 2 Subjects from Mexican Southeastern: A Cross Sectional Study
Source: Medicina (Kaunas). 2023 Aug 11;59(8):1451. doi: 10.3390/medicina59081451 (PMC10456706; doi:10.3390/medicina59081451)
Supplement: Supplementary file 1 [file medicina-59-01451-s001.zip › Table S2 suppl.pdf]

Table S2. ALB-g, HbA1c, and PTD correlations in DM (n=66) and noDM (n=74) women.

		DM2		noDM		DM2		noDM		DM2		noDM	
		PTD				ALB-g				HbA1c			
		r	p	r	p	r	p	r	p	r	p	r	p
BMD (g/cm^2)	Total	**-0.367**	**0.042**	0.291	0.141	-0.249	0.177	0.079	0.691	-0.317	0.083	0.193	0.326
	Arms	**-0.389**	**0.031**	0.294	0.137	-0.181	0.330	0.156	0.427	**-0.514**	**0.003**	0.264	0.175
	Ribs	**-0.452**	**0.011**	0.234	0.240	-0.297	0.104	0.201	0.304	-0.204	0.272	0.353	0.066
	Spine	**-0.475**	**0.007**	0.208	0.298	-0.18	0.332	-0.145	0.463	-0.186	0.317	-0.266	0.172
	Pelvis	**-0.511**	**0.003**	0.223	0.263	-0.119	0.524	0.197	0.314	0.204	0.272	-0.15	0.446
	Legs	**-0.462**	**0.009**	0.348	0.075	-0.230	0.213	0.244	0.211	-0.353	0.051	0.056	0.777
BMC (g)	Total	**-0.461**	**0.009**	0.282	0.155	**-0.434**	**0.015**	0.053	0.787	**-0.428**	**0.016**	0.018	0.927
	Arms	**-0.424**	**0.017**	0.243	0.222	**-0.372**	**0.039**	0.230	0.239	**-0.476**	**0.007**	-0.098	0.621
	Ribs	**-0.457**	**0.010**	0.313	0.111	-0.12	0.520	0.158	0.421	-0.21	0.258	0.336	0.081
	Spine	**-0.601**	**0.001**	-0.054	0.790	-0.278	0.130	-0.181	0.356	-0.194	0.295	-0.289	0.136
	Pelvis	**-0.418**	**0.019**	0.239	0.229	-0.234	0.205	0.156	0.427	-0.207	0.265	-0.203	0.299
	Legs	**-0.522**	**0.003**	0.343	0.080	-0.341	0.061	0.244	0.210	**-0.444**	**0.012**	-0.081	0.681
Fat mass (g))	Total	**-0.404**	**0.024**	0.083	0.680	-0.103	0.580	-0.276	0.155	0.290	0.113	-0.070	0.725
	Arms	-0.207	0.264	0.104	0.606	-0.046	0.808	-0.135	0.494	0.254	0.168	-0.09	0.648
	Trunk	**-0.513**	**0.003**	0.084	0.676	-0.178	0.337	-0.321	0.096	0.246	0.182	-0.169	0.390
	Legs	**-0.371**	**0.040**	0.087	0.665	-0.185	0.319	-0.170	0.388	0.351	0.053	-0.113	0.568
Fat mass (%)	Total	-0.232	0.209	-0.029	0.887	-0.203	0.273	-0.089	0.653	**0.447**	**0.012**	-0.348	0.069
	Arms	-0.080	0.670	-0.091	0.652	-0.183	0.325	-0.065	0.742	**0.386**	**0.032**	-0.215	0.271
	Trunk	-0.255	0.165	-0.022	0.913	-0.158	0.396	-0.062	0.753	**0.381**	**0.035**	-0.473	0.051
	Legs	-0.234	0.205	-0.241	0.225	-0.196	0.291	-0.099	0.616	**0.540**	**0.002**	-0.226	0.248
Anthropometry Indexes	BMI	**-0.403**	**0.025**	0.027	0.894	-0.033	0.861	-0.247	0.206	0.285	0.121	0.128	0.517
	W/Ht	**-0.374**	**0.038**	0.124	0.538	0.009	0.963	-0.076	0.702	0.299	0.102	0.271	0.164
	RFM	-0.272	0.138	-0.231	0.247	-0.059	0.753	-0.267	0.170	0.331	0.069	-0.066	0.739
	W/H	-0.279	0.129	0.377	0.052	0.228	0.218	0.137	0.488	0.170	0.360	0.371	0.052
	Waist girth	**-0.483**	**0.006**	0.218	0.274	-0.122	0.513	-0.098	0.62	0.096	0.609	0.196	0.317
	Hip girth	**-0.364**	**0.044**	-0.031	0.877	-0.173	0.353	-0.373	0.051	0.099	0.596	-0.094	0.633
	Neck girth	**-0.429**	**0.016**	0.174	0.387	-0.088	0.639	-0.37	0.053	0.004	0.982	-0.141	0.473

DM2= Diabetes mellitus 2, noDM=No diabetes mellitus, PTD=Pentosidine, ALB-g= glycated Albumin, HbA1c= glycated hemoglobin, BMD= Bone mineral density, BMC=Bone mineral content, RFM= Relative fat mass, BMI= Body mass index, W/H= Waist-to-Hip ratio, W/Ht= Waist-to-height ratio.
